# NURSING HOME PALLIATIVE CARE DURING THE PANDEMIC: DIRECTIONS FOR THE FUTURE

**DOI:** 10.1093/geroni/igac059.2368

**Published:** 2022-12-20

**Authors:** Kacy Ninteau, Christine Bishop

**Affiliations:** Dana Farber Cancer Institute, Boston, Massachusetts, United States; Brandeis University, Waltham, Massachusetts, United States

## Abstract

For this qualitative, pilot study, seven Massachusetts nursing home Directors of Nursing (DONs) were interviewed remotely about palliative care provision before and during the COVID-19 pandemic. Interview data were analyzed using thematic analysis. Palliative care addresses physical, emotional, psychological, and spiritual suffering that accompanies serious illness. Symptom management and goals of care is especially valuable for seriously ill nursing home residents. We investigated the solutions nursing home staff developed to provide palliative care during the COVID-19 pandemic despite restrictions on external resources. Before the pandemic, palliative care was delivered primarily by nursing home staff depending on formal and informal consultations from palliative care specialists affiliated with hospice providers. When COVID-19 lockdowns precluded these consultations, nursing staff did their best to provide palliative care, but were often overwhelmed by shortfalls in resources, resident decline brought on by isolation and COVID-19 itself, and a sense that their expertise was lacking. Advance care planning conversations focused on hospitalization status and options for care given COVID-19 resource constraints. Nevertheless, nursing staff discovered previously untapped capacity to provide palliative care on-site as part of standard care, building trust of residents and families. Nursing staff rose to the palliative care challenge during the COVID-19 pandemic, albeit with great effort nursing home payment and quality standards should support development of in-house staff capacity to deliver palliative care while expanding access to the formal consultations and family involvement that were restricted by the pandemic.

